# Supplementary material for: Organic laser power converter for efficient wireless micro power transfer
Source: Nat Commun. 2023 Sep 7;14:5511. doi: 10.1038/s41467-023-41270-1 (PMC10484967; doi:10.1038/s41467-023-41270-1)
Supplement: Supplementary file 1 — Supplementary Information [file 41467_2023_41270_MOESM1_ESM.pdf]

Supplementary Information for

Organic Laser Power Converter for Efficient Wireless Micro Power Transfer

Yafei Wang[1,2], Zhong Zheng[1,3*], Jianqiu Wang[1], Pengqing Bi[1], Zhihao Chen[1], Junzhen Ren[1,2], Cunbin An[1], Shaoqing Zhang[1,3] and Jianhui Hou[1,2,3*]

[1]State Key Laboratory of Polymer Physics and Chemistry, Beijing National Laboratory for Molecular Sciences, Institute of Chemistry, Chinese Academy of Sciences, Beijing 100190, China.

[2]University of Chinese Academy of Sciences, Beijing 100049, China

[3]School of Chemistry and Biology Engineering, University of Science and Technology Beijing, Beijing 100083, China.

**corresponding author email: zhongzheng@ustb.edu.cn; hjhzlz@iccas.ac.cn*

Keywords organic photovoltaics, laser power converter, wireless power transfer, power conversion efficiency, laser charge

The PDF file includes:

Supplementary Note 1. The various applications of laser wireless power transfer at different laser wavelengths and output powers.

Supplementary Note 2. Lasers spectrum line shapes.

Supplementary Note 3. The photovoltaic parameters of PBDB-TF:BTP-eC9 under AM 1.5G and Lasers.

Supplementary Note 4. The Supplementary data of transient absorption (TA).

Supplementary Note 5. Exciton diffusion constants measurements and calculation.

Supplementary Note 6. Temperature dependent photovoltaic parameters.

Supplementary Note 7. Predicted photovoltaic parameters.

Supplementary Note 8. Device fabrication and characterization.

Supplementary Note 9. Comparison of performance between GaAs, PVK, Si and OLPC under laser illumination.

Supplementary Note 1. The various applications of laser wireless power transfer at different laser wavelengths and output powers.

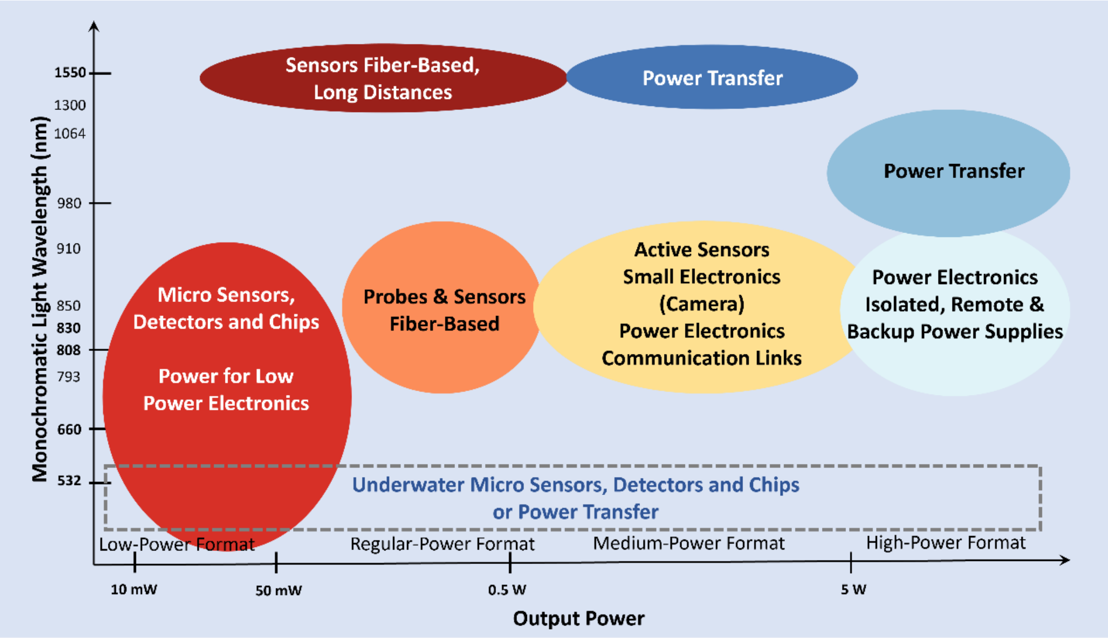

Supplementary Fig. 1. Illustration of the various WPT applications organized according to the output power (horizontal axis—log scale) and the wavelength of monochromatic (vertical axis—not to scale). The commercial aspects, the reliability, and the technical attributes of the available laser diode products often predominantly guide the selection of the optical input wavelength. We classify the LPC devices into low/regular/medium/high power, based on their output power capabilities.[1]

As shown in **Supplementary Fig. 1** Some micro sensors, detectors and chips only require a few milliwatts or even microwatts of power per use, and they do not potentially require a long-term power supply, such as anti-theft or anti-counterfeiting keys, bank cards (or other important cards), passive electronic tags (the ink screen does not consume power during daily display) and onboard ETC etc. Except for these consumer electronics, the energy consuming of long-term underwater detectors are usually as low as milliwatt-scale. The charge for these electronics could be convenient when using power transfer. Therefore, the importance of developing laser power converter suitable for 10^{-1} to 10^{2} mW cm^{-2} (Low power) WPT will be unfolded rapidly as the applications of IoT expand.

Supplementary Note 2. Lasers spectrum line shapes.

The spectrum line shapes of the lasers used in this work were shown in Figure S1, the Lorentz line shapes has been revealed by the good coincidence between experimental intensity distribution $L(\omega\text{-}\omega_0)$ and the normalized Lorentz model:

$$L(\omega-\omega_0)=\frac{\gamma}{2\pi(\omega-\omega_0)^2+2\pi(\gamma/2)^2} \qquad \text{Supplementary Equation (1)}$$

where ω_0 is central angular frequency, ω is angular frequency and γ is damping rate which equals to the full width at half maxima (FWHM). The small FWHMs in line shapes of λ_{532}, λ_{660}, and λ_{809} demonstrate the reasonability of treating the used lasers as absolute monochromatic lights.

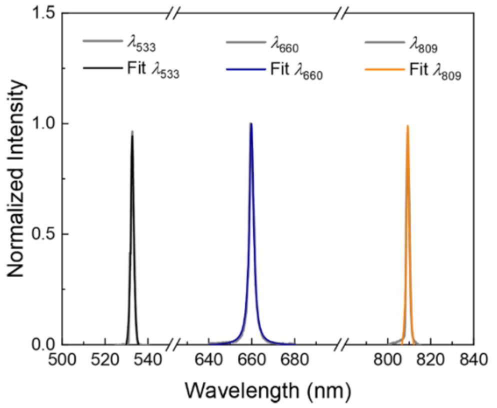

Supplementary Fig. 2. The spectrum line shapes of the lasers.

Supplementary Note 3. The photovoltaic parameters of PBDB-TF:BTP-eC9 under AM 1.5G and Lasers.

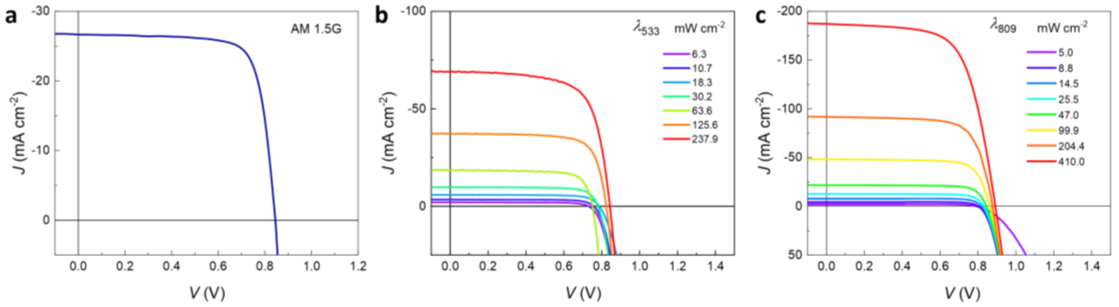

Supplementary Fig. 3. The *J*-*V* curves of PBDB-TF:BTP-eC9 with **a.** AM 1.5G **b.** 533 nm and **c.** 809 nm Lasers.

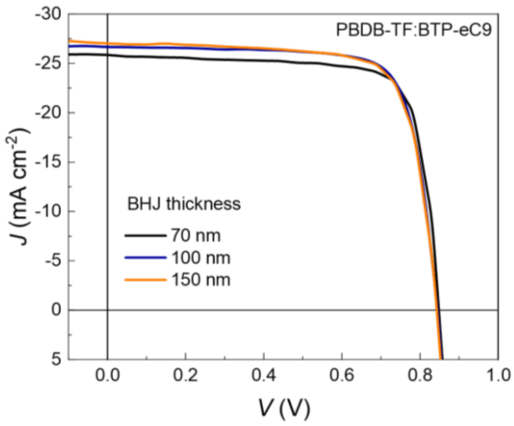

Supplementary Fig. 4. The *J*-*V* curves of PBDB-TF:BTP-eC9 with AM 1.5G and different film thickness.

Supplementary Table 1. The photovoltaic parameters of PBDB-TF:BTP-eC9 with AM 1.5G and Lasers.

Light source	I_0 (mW cm^{-2})	V_{oc} (V)	J_{sc} (mA cm^{-2})	FF (%)	PCE (%)	EQE (%)
AM 1.5G	100.0	0.845	26.6	77.4	17.4	-
	6.3	0.730	2.1	74.5	17.7	75.9
	10.7	0.750	3.4	75.8	18.1	73.9
	18.3	0.770	5.8	76.6	18.9	74.5
λ_{533}	30.2	0.780	9.8	76.5	19.4	75.5
	63.6	0.800	19.8	76.0	18.9	72.3
	125.6	0.820	37.3	74.6	18.1	69.1
	237.9	0.840	69.1	69.1	16.9	67.5
	5.0	0.790	2.3	76.6	28.3	70.2
	8.8	0.800	4.5	79.2	32.4	76.7
	14.5	0.820	7.6	79.5	33.9	78.0
λ_{809}	25.5	0.830	12.7	79.0	32.8	75.0
	47.0	0.840	21.8	78.4	30.5	69.4
	99.9	0.860	44.1	76.9	29.2	66.2
	204.4	0.880	91.7	72.4	28.6	67.2
	410.0	0.890	187.1	66.1	26.8	68.4

Supplementary Note 4. The Supplementary data of transient absorption (TA).

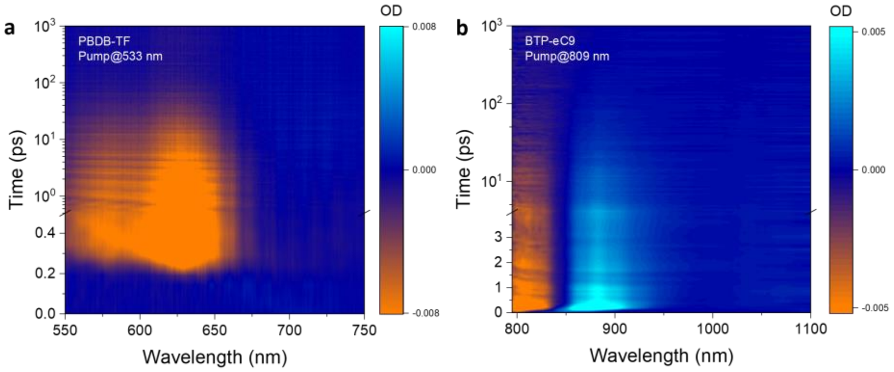

Supplementary Fig. 5. The TA 2D-images of PBDB-TF and BTP-eC9 neat films at **a.** 533 and **b.** 809 nm Laser.

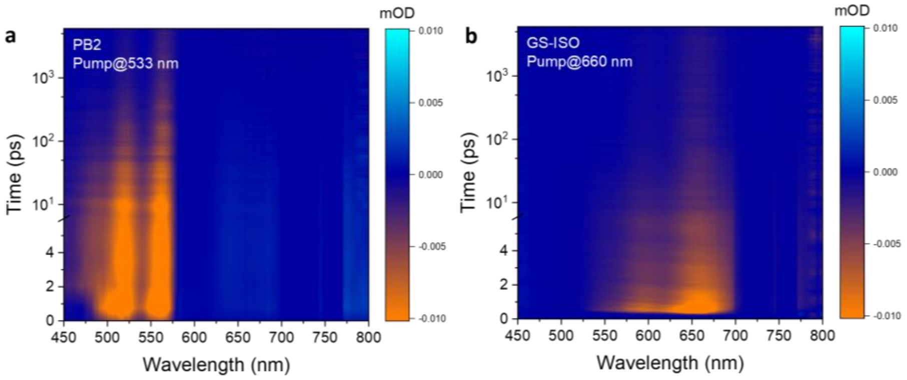

Supplementary Fig. 6. The TA 2D-images of PB2and GS-ISO neat films at of **a.** 533 and **b.** 660 nm Laser.

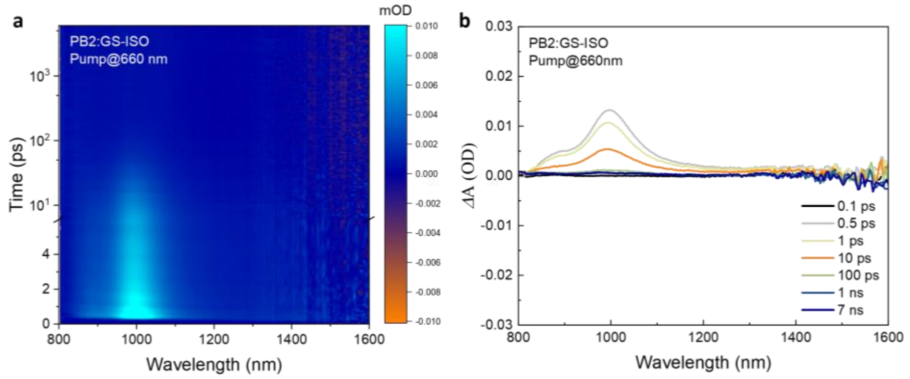

Supplementary Fig. 7. a. The transient absorption (TA) two dimensional images of PB2:GS-ISO BHJ under excitation of 660 nm, 10.0 μJ cm^{-2}. **b.** The TA spectra at different delay times of PB2:GS-ISO film with excitation at 660 nm.

Supplementary Table 2. Lifetime of the electron and hole transfer process in OLPC. The data was achieved through biexpoential fitting.

BHJ	Excitation wavelength (nm)	A_1	τ_1 (ps)	A_2	τ_2 (ps)
PBDB-TF: BTP-eC9	533	0.27±0.08	0.97±0.04	0.51±0.09	24.06±0.11
	809	0.38±0.05	1.68±0.03	0.13±0.03	26.45±0.59
PB2:GS-ISO	533	0.54±0.02	1.76±0.12	0.20±0.01	186.65±23.78
	660	0.82±0.03	0.99±0.07	0.33±0.02	10.60±0.78

Supplementary Note 5. Exciton diffusion constants measurements and calculation.

The singlet-singlet exciton annihilation (SSA) method[2] is used to measure exciton diffusion constants. The femtosecond TA spectroscopy was employed to measure exciton lifetimes as a function of excitation density. The excitation fluences range for PBDB-TF and BTP-eC9 neat films from 0.2 μJ cm^{-2}, 1.8 μJ cm^{-2}, 3.5 μJ cm^{-2}, 8.0 μJ cm^{-2} up to 13.0 μJ cm^{-2}. While assuming annihilation destroys both excitons, there are two main quenching channels for excitons, bimolecular (exciton annihilation) and monomolecular decay pathways. The thicknesses of PBDB-TF and BTP-eC9 is 98 and 103 nm, respectively. The relationship is shown as follows:

$$-\frac{dn(t)}{dt} = kn(t) + \gamma n^2(t) \qquad \text{Supplementary Equation (2)}$$

$$n(t) = \frac{n(0)\exp(-kt)}{1 + \frac{\gamma}{k} n(0)[1 - \exp(-kt)]} \qquad \text{Supplementary Equation (3)}$$

where, $n(t)$ is the singlet exciton density at a decay time of t, k is the monomolecular decay rate and γ is the singlet-singlet bimolecular exciton annihilation rate. We assume that EEA is completely absent at low excitation intensity and at high excitation intensities, where EEA takes place.

At a low excitation intensity of 0.8 μJ cm^{-2}, as expected, the decay dynamics are approximately monoexponential (To avoid bimolecular recombination, polystyrene was added to the film.), which obey the following equations:

$$\frac{dn(t)}{dt} = -kn(t) \qquad \text{Supplementary Equation (4)}$$

$$t_{\frac{1}{2}} = t_0 = \frac{\ln 2}{k} \qquad \text{Supplementary Equation (5)}$$

where $t_{\frac{1}{2}}$ is the half-life of total photoinduced exciton, the time taken for the concentration of exciton to decay to half its initial value. On the other hand, EEA effect occurs when a relatively high excitation intensity is used, which leads to obviously different decay dynamics, and we can obtain the equations as follows:

$$t_{\frac{1}{2}} = \frac{\alpha ln2}{k}, \alpha < 1 \qquad \text{Supplementary Equation (6)}$$

$$\gamma = \frac{k(2\exp(-\alpha \ln 2) - 1)}{n_0(1 - \exp(-\alpha \ln 2))} \quad \text{Supplementary Equation (7)}$$

If the value of α is low enough, we can consider that $\exp(-\alpha \ln 2) = 1 - \alpha \ln 2$. Then the above equation can be equivalent to:

$$\gamma = \frac{k(2 - 2\alpha \ln 2 - 1)}{n_0 \alpha \ln 2} \quad \text{Supplementary Equation (8)}$$

When the excitation power density is 30 μJ cm^{-2}, we can that the calculated values of γ are 1.18×10^{-9} and 2.00×10^{-9} cm^3 s^{-1} for PBDB-TF and BTP-eC9. Then, the diffusion coefficient D can be obtained through the following equation:

$$D = \frac{\gamma}{4\pi R} \quad \text{Supplementary Equation (9)}$$

where R is the annihilation radius of singlet excitons. R is assumed to be 2 nm.

Supplementary Table. 3. Detailed parameters of single exciton decay dynamic for PBDB-TF and BTP-eC9.

Materials	Excitation energy (μJ cm^{-2})	$n_0\times10^{17}$ (cm^{-3})	$t_{1/2}$ (ps)	$k\times10^{-9}$ (s^{-1})	α	$\gamma\times10^{-9}$ (cm^3 s^{-1})	D (cm^2 s^{-1})	T (ps)	L_D (nm)
PBDB-TF	0.8	3.36	131	5.30	0.10	-	-	-	-
	13.0	55.54	-	-	-	1.79	0.71	103	27
BTP-eC9	0.8	3.08	578	1.20	0.10	-	-	-	-
	13.0	52.04	-	-	-	2.50	0.99	97	31

Supplementary Note 6. Temperature dependent photovoltaic parameters.

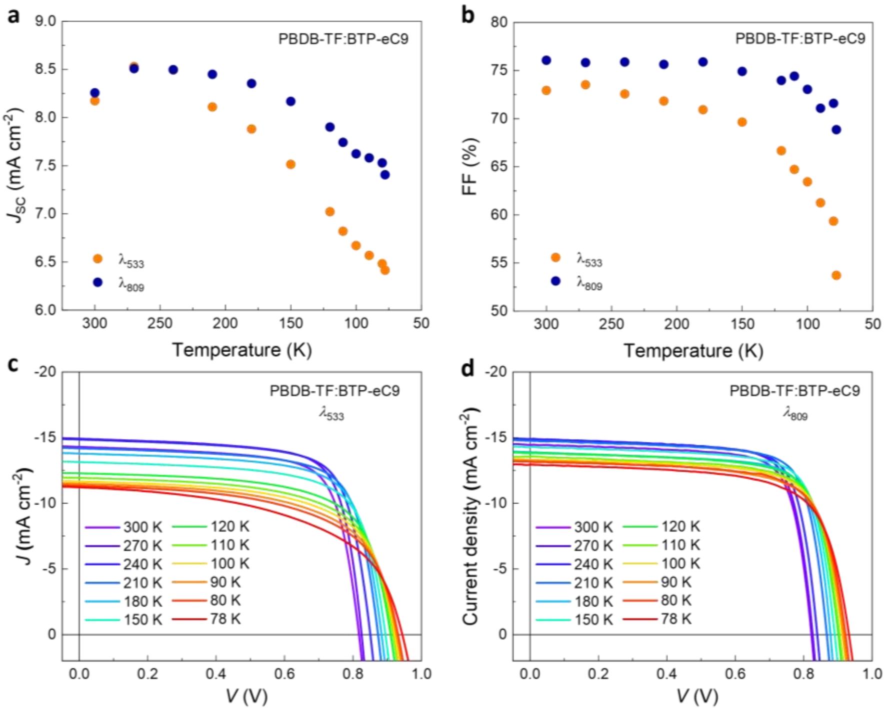

Supplementary Fig. 8. Temperature dependent **a.** J_{sc}, **b.** FF and the *J-V* carves of PBDB-TF:BTP-eC9 based solar cell with different illumination intensity at **c.** 533 and **d.** 809 nm laser.

The temperature-dependent EQE values for all the devices can be fitted using the following equation[3]:

$$\mathrm{EQE} = EQE_0 exp\left(-\frac{E_a}{k_B T}\right) \quad \text{Supplementary Equation (10)}$$

$$\ln\mathrm{EQE} = \ln EQE_0 - \frac{E_a}{k_B \mathrm{T}} \quad \text{Supplementary Equation (11)}$$

where EQE_0 is the EQE value at infinite temperature, E_a is the activation energy, k_B is the Boltzmann constant, and T is the temperature. The E_a value indicates the energy required for the geminate pair separation. Therefore, the E_a can be fitted with temperature dependent *J-V* carves.

Supplementary Table 4. Temperature dependent photovoltaic parameters of PBDB-TF:BTP-eC9 based solar cell with different illumination intensity at 533 nm laser (26.8 mW cm^{-2}).

Temperature (K)	V_{oc} (V)	J_{sc} (mA cm^{-2})	FF (%)	PCE (%)
300	0.818	8.2	72.9	18.2
270	0.825	8.5	73.5	19.3
240	0.851	8.5	72.6	19.6
210	0.873	8.1	71.8	19.0
180	0.884	7.9	70.9	18.4
150	0.895	7.5	69.7	17.5
120	0.910	7.0	66.7	15.9
110	0.917	6.8	64.7	15.1
100	0.921	6.7	63.5	14.5
90	0.927	6.6	61.3	13.9
80	0.932	6.5	59.4	13.4
78	0.944	6.4	53.7	12.1

Supplementary Table 5. Temperature dependent photovoltaic parameters of PBDB-TF:BTP-eC9 based solar cell with different illumination intensity at 809 nm laser (17.2 mW cm^{-2}).

Temperature (K)	V_{oc} (V)	J_{sc} (mW cm^{-2})	FF (%)	PCE (%)
300	0.822	8.3	76.1	30.2
270	0.826	8.5	75.8	30.9
240	0.840	8.5	75.9	31.5
210	0.867	8.5	75.7	32.4
180	0.878	8.4	75.9	32.5
150	0.891	8.2	74.9	31.8
120	0.901	7.9	74.00	30.6
110	0.904	7.7	74.4	30.1
100	0.912	7.6	73.1	29.5
90	0.916	7.6	71.1	28.8
80	0.922	7.5	71.6	28.8
78	0.932	7.4	68.9	27.6

Supplementary Note 7. Predicted photovoltaic parameters.

On the basis of previous theoretical work and state-of-the art experimental results, we first have carried out a semiempirical analysis for the possible but realistic PCE limit of OLPC under Air Mass monochromatic light wavelength[4].

Base the basic assumption for the Shockley-Queisser (SQ) limit, the power conversion efficiency (PCE) indicates the part of the incident light energy converting into effective electrical energy, namely, the ratio of the maximum output power density (P_{max}) to the incident light power density (I_0), as follows:

$$\mathrm{PCE} = \frac{P_{\max}}{I_0} \times 100\% = \frac{V_{oc} \times J_{sc} \times \mathrm{FF}}{I_0} \times 100\% \qquad \text{Supplementary Equation (12)}$$

where V_{oc} is the open-circuit voltage, J_{sc} is the short-circuit current density, FF is the fill factor, the fundamental assumptions are made as follows:

(i) the V_{oc} is determined by the equation $eV_{oc}=E_{MIL}–V_{loss}$, where $E_{MLW}=1240/\lambda$ (λ is the wavelength of monochromatic light, and the λ is selected according to the absorption of the BHJ), V_{loss} is the energy loss, e is the elementary charge;

(ii) the J_{sc} is assumed to be the theoretical current in the entire BHJ absorption range multiplied by a given external quantum efficiency (EQE) value, as follows:

$$J_{sc} = \mathrm{e}\int_0^{\lambda} \mathrm{N}_{\lambda}(\lambda)\mathrm{EQE}(\lambda)\mathrm{d}\lambda) \qquad \text{Supplementary Equation (13)}$$

(iii) the FF is assumed to be the semi-empirical equation[5,6]

$$\mathrm{FF} = \frac{\mathrm{v}_{oc} - \ln(\upsilon_{oc} + 0.72)}{\upsilon_{oc} + 1} - 0.1 \qquad \text{Supplementary Equation (14)}$$

where the v_{oc} ($=\frac{ev_{oc}}{nk_BT}, n = 1$) is the normalized open-circuit voltage (n is the diode ideal factor, k_B is the Boltzmann constant, and T is the temperature.)

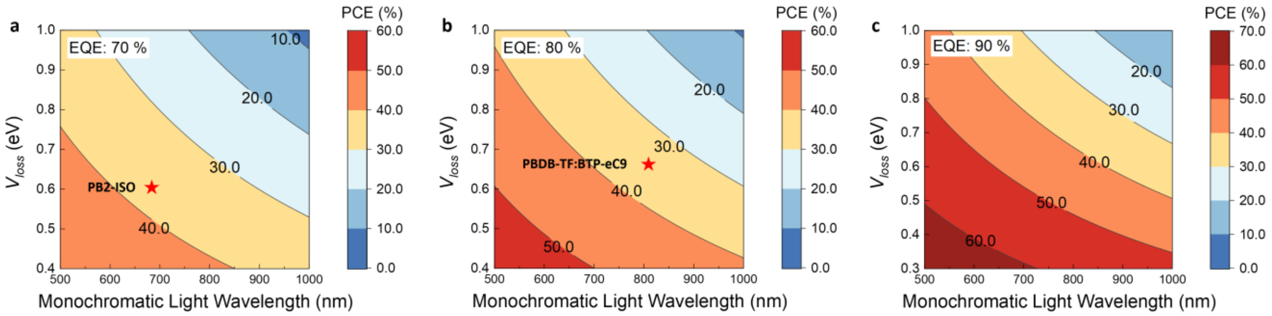

Supplementary Fig. 9. The predicted achievable PCEs of OLPC under the monochromatic light wavelength from 500 to 1000 nm, V_{loss} is the value of 0.5 eV, EQE is **a**. 70% **b**. 80% and **c**. 90%.

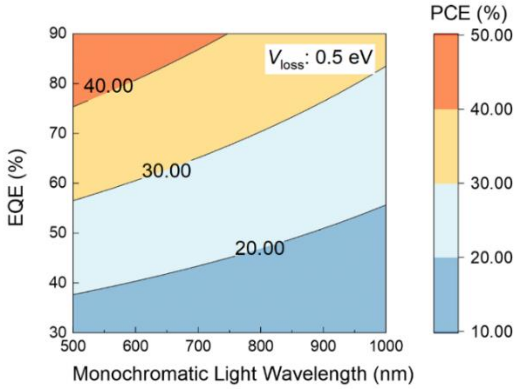

Supplementary Fig. 10. Predicted photovoltaic parameters of solar cells based on semi-empirical analysis under monochromatic light wavelength. The PCEs versus monochromatic light wavelengths and EQE, assuming V_{loss} of the monochromatic light wavelengths is 0.5 eV.

Supplementary Note 8. Device fabrication and characterization.

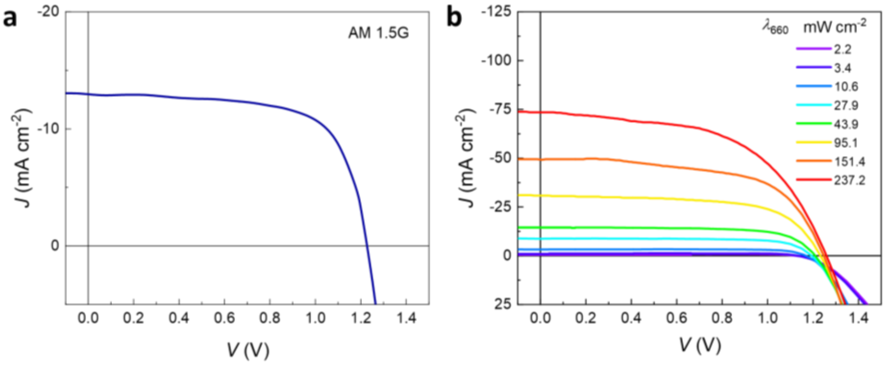

Supplementary Fig. 11. The *J*-*V* curves of PB2:GS-ISO-based OLPC under **a.** AM 1.5G and **b.** 660 nm Laser.

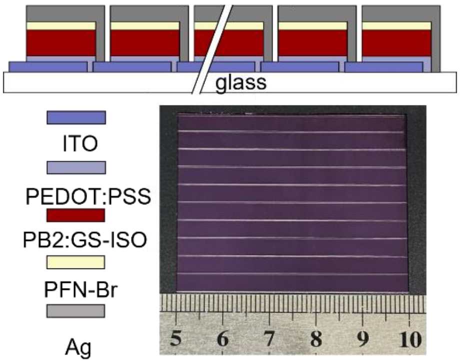

Supplementary Fig. 12. Schematic diagram of 20 cm^2 module.

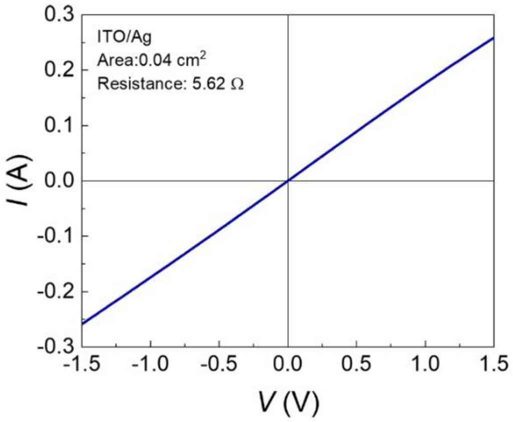

Supplementary Fig. 13. The resistance of ITO/Ag of the devices with area of 0.04 cm^2, which tested by *J*-*V* curve.

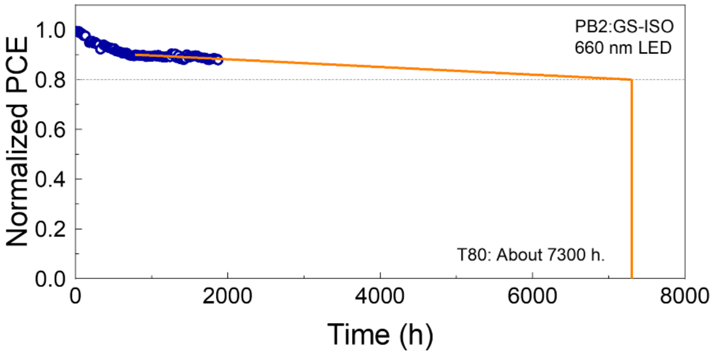

Supplementary Fig. 14. The stability of PB2:GS-ISO of 9.5 mW cm^{-2} with 660 nm LED.

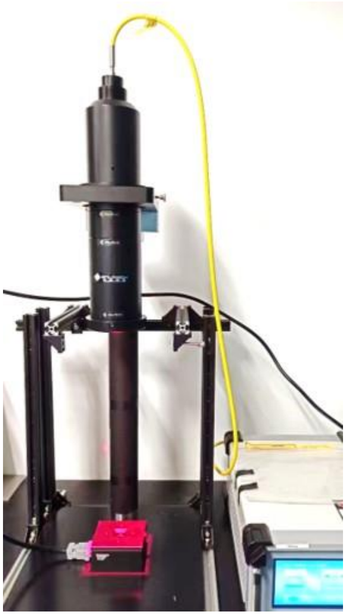

Supplementary Fig. 15. The test system of OLPC.

Supplementary Note 9. Comparison of performance between GaAs, PVK, Si and OLPC under laser illumination.

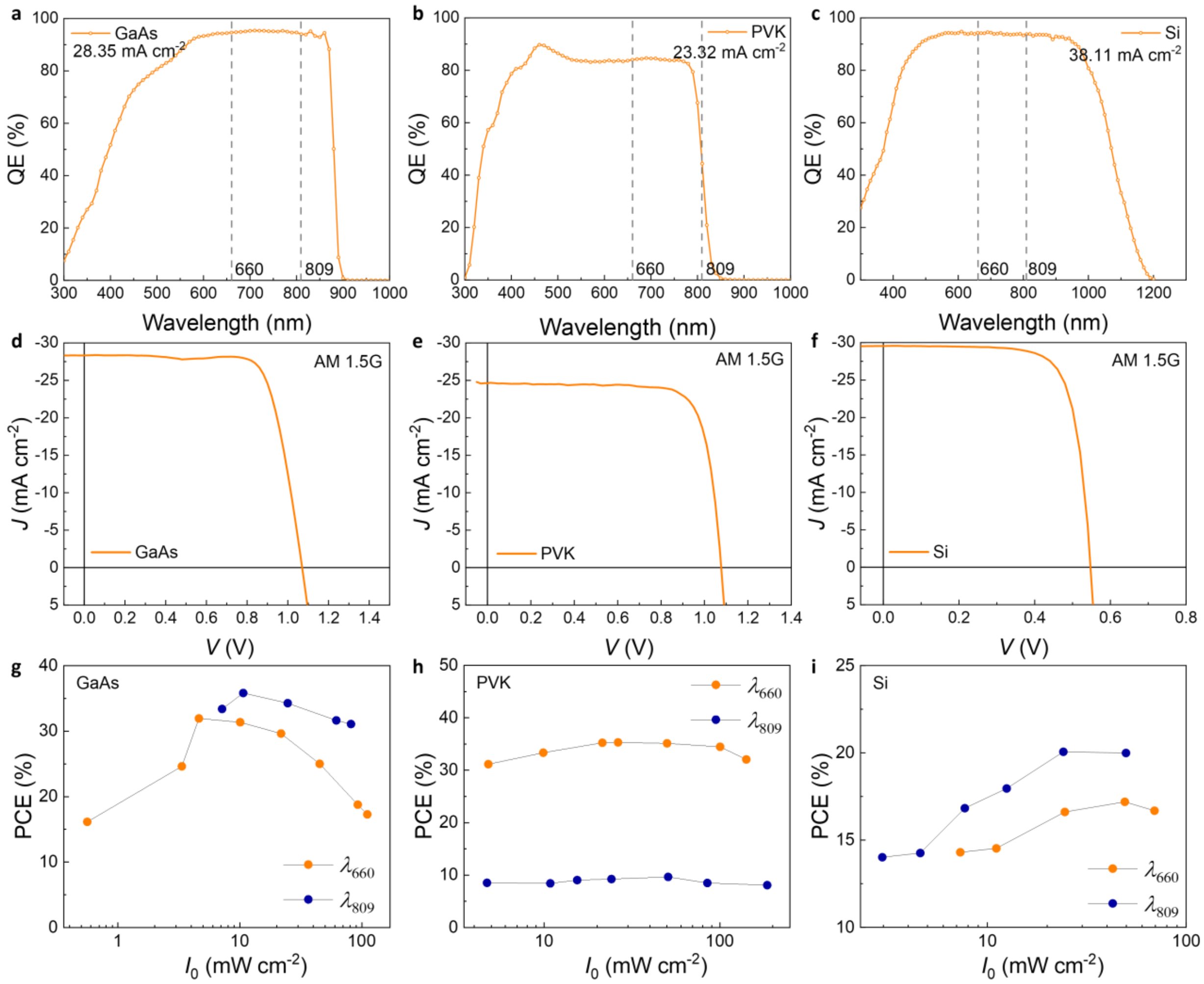

Supplementary Fig. 16. The EQE spectra of **a.** Gallium arsenide (GaAs), **b.** perovskite (PVK) and **c.** silicon (Si) under the illumination of AM 1.5G 100 mW m^{-2}. The *J-V* curves of **d.** GaAs, **e.** PVK and **f**. Si with AM 1.5G. The I_0-dependent PCE of the **g.** GaAs, **h.** PVK and **i.** Si under lasers with different wavelengths. The lasers are labeled by λ with wavelength marked in subscript. The specific parameters in the figures are attached in the part I of the attachment.

Supplementary Table 6. Performance and cost of photoelectric converters.

**Photovoltaic converter**	**Bandgap adjustment**	**Flexibility** [a]	**Heavy Metal** [b]	**Power per weight (AM 1.5G)** [c] (W g^{-1})	**Power per weight (Laser)** [d] (mW g^{-1})	**Cost (CNY cm^{-2})**
GaAs	Limited	+	○	1.0 [e]	1.3	65.6 [f]
				3.0 [7]	3.9	
Si	Limited	+	×	~0.3 [8]	0.4	0.06 [g]
PVK	Limited	++	○	30.3 [9]	49.5	2.0 [h]
OLPC	High	+++	×	40.7 [10]	83.7	4.0 [i]

[a]: The more the number of "+", the better the flexibility of photovoltaic converters.

[b]: "○" indicates the presence of heavy metal, while "×" indicate the absence of heavy metal.

[c]: The power per weight of photoelectric converters reported in the literatures at 100 mW cm^{-2}.

[d]: The power per weight of photoelectric converters are calculated by the value of literatures, and the illumination intensity of the lasers assumed 10 mW cm^{-2}.[7-10] Due to the lower PCE of commercial cells, the PCEs of GaAs and Si cells are calculated based on the laboratory's PCEs.

[e]: Provided by merchants.

[f, g]: Provided by merchants, calculated based on the purchase price.

[h]: The cost of preparing PVK devices, and the details are shown in **Supplementary Table 10.**

[i]: Literature report.

As shown in **Supplementary Fig. 16**, in the view point of PCE under low photon flux, GaAs and perovskite LPC (laser power convertor) exhibit better performance than OLPC (organic laser power convertor); while the Si LPC shows even lower PCE than OLPC. Nevertheless, OLPC is still indispensable owing to the absolute advantage in energy budget and laser wavelength adaptability. Based on detail investigation, we summarized the bandgap tunability, flexibility, contain heavy metals or not, power per weight and cost in **Supplementary Table 6**. Due to the adjustable bandgap, flexibility, absence of heavy metals, high power per weight, and low cost, OLPCs have unique advantages in wireless power transfer.

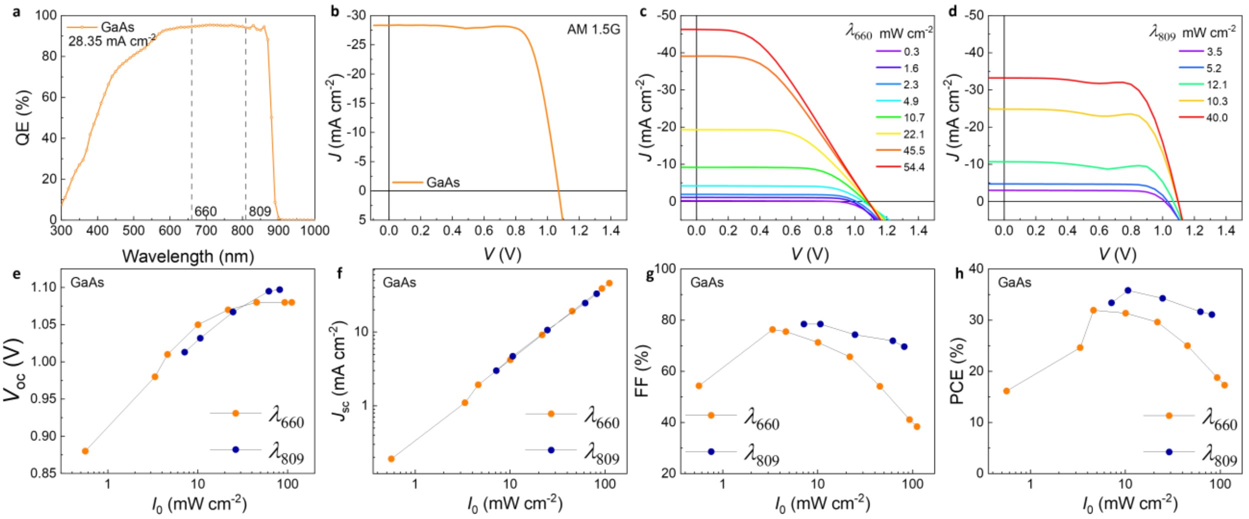

Supplementary Fig. 17. a. The EQE spectra of GaAs under the illumination of AM 1.5G 100 mW m^{-2}. **b~d.** The *J*-*V* curves of GaAs with AM 1.5G and Lasers. **e~h.** The I_0-dependent V_{oc}, J_{sc}, FF and PCE of the GaAs under lasers with different wavelengths. The lasers are labeled by λ with wavelength marked in subscript.

Supplementary Table 7. The photovoltaic parameters of GaAs with AM 1.5G and Lasers.

Light source	I_0 (mW cm^{-2})	V_{oc} (V)	J_{sc} (mA cm^{-2})	FF (%)	PCE (%)
AM 1.5G	100.0	1.070	28.3	75.5	22.9
λ_{660}	0.6	0.880	0.2	54.3	16.1
	3.3	0.980	1.1	76.3	24.6
	4.6	1.010	1.9	75.5	31.9
	10.1	1.050	4.2	71.3	31.4
	21.8	1.070	9.2	65.7	29.6
	45.2	1.080	19.3	54.1	25.0
	92.8	1.080	39.1	41.1	18.8
	111.1	1.080	46.3	38.4	17.3
λ_{809}	7.1	1.013	3.0	78.5	33.4
	10.7	1.032	4.7	78.5	35.8
	24.7	1.067	10.7	74.3	34.3
	61.8	1.095	24.8	71.9	31.6
	81.6	1.097	33.2	69.6	31.1

*: The Si is commercial cell, and the area of the cell is 9.9 cm^2.

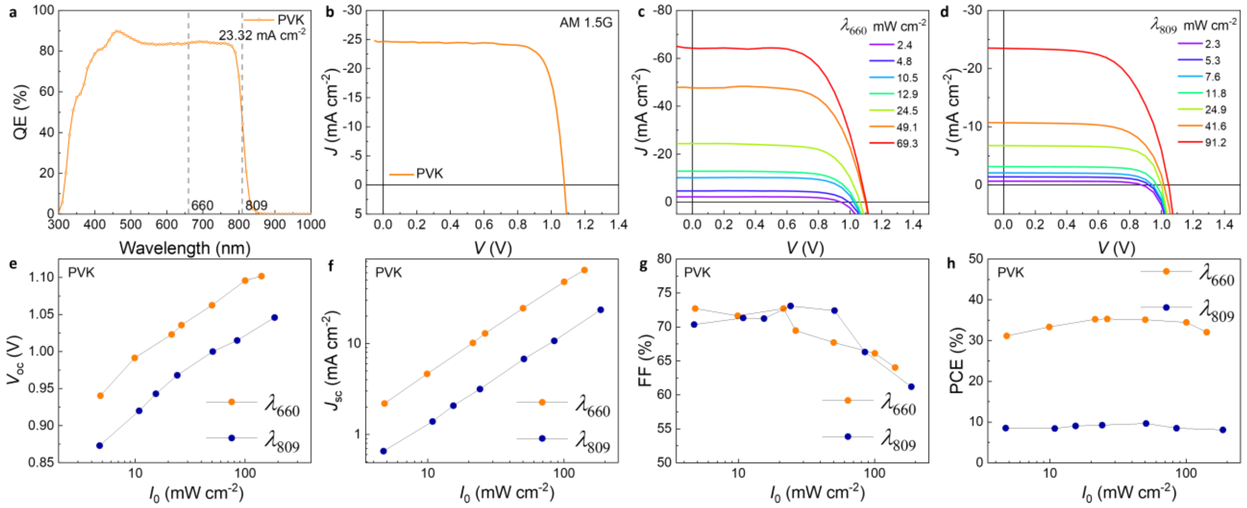

Supplementary Fig. 18. a. The EQE spectra of PVK under the illumination of AM 1.5G 100 mW m^{-2}. **b~d.** The *J-V* curves of PVK with AM 1.5G and Lasers. **e~h.** The I_0-dependent V_{oc}, J_{sc}, FF and PCE of the PVK under lasers with different wavelengths. The lasers are labeled by λ with wavelength marked in subscript.

Supplementary Table 8. The photovoltaic parameters of PVK with AM 1.5G and Lasers.

Light source	I_0 (mW cm^{-2})	V_{oc} (V)	J_{sc} (mA cm^{-2})	FF (%)	PCE (%)
AM 1.5G	100.0	1.080	24.6	78.3	20.8
	4.8	0.940	2.2	72.7	31.2
	9.9	0.990	4.6	71.7	33.3
	21.4	1.020	10.2	72.7	35.2
λ_{660}	26.3	1.040	12.9	69.5	35.3
	50.1	1.060	24.4	67.7	35.1
	100.3	1.100	47.7	66.1	34.5
	141.3	1.100	64.2	64.0	32.1
	4.7	0.873	0.7	70.4	8.5
	10.8	0.920	1.4	71.3	8.4
	15.4	0.943	2.1	71.2	9.0
λ_{809}	24.1	0.968	3.2	73.1	9.2
	50.8	1.000	6.8	72.4	9.6
	84.8	1.015	10.7	66.3	8.5
	186.0	1.046	23.5	61.2	8.1

*: The area of the PVK cell is 0.04 cm^2.

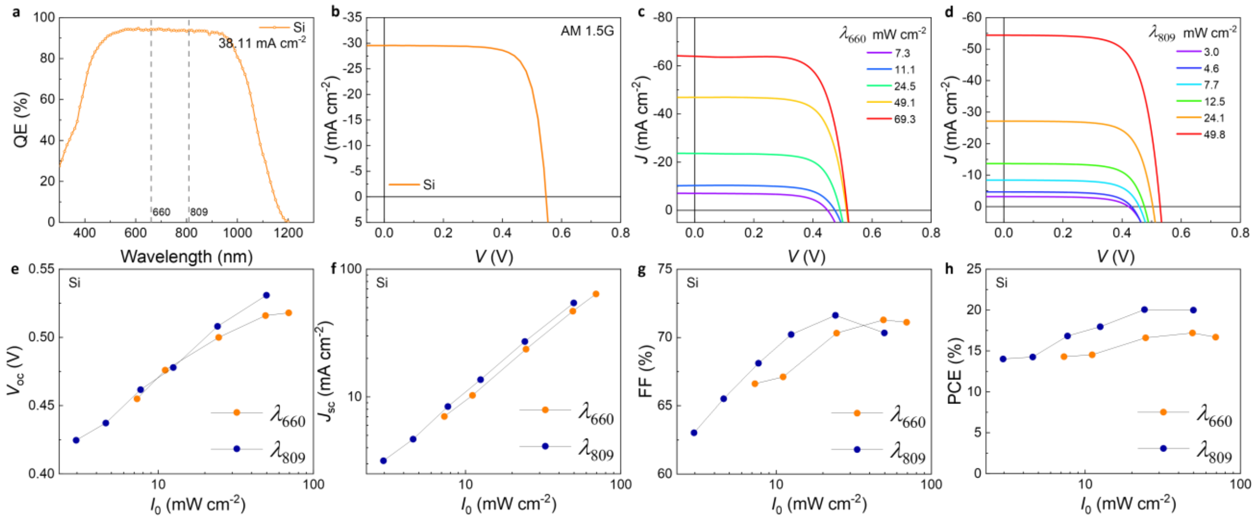

Supplementary Fig. 19. a. The EQE spectra of Si under the illumination of AM 1.5G 100 mW m^{-2}. **b~d.** The *J-V* curves of Si with AM 1.5G and Lasers. **e~h.** The I_0-dependent V_{oc}, J_{sc}, FF and PCE of the Si under lasers with different wavelengths. The lasers are labeled by *λ* with wavelength marked in subscript.

Supplementary Table 9. The photovoltaic parameters of Si with AM 1.5G and Lasers.

Light source	I_0 (mW cm^{-2})	V_{oc} (V)	J_{sc} (mA cm^{-2})	FF (%)	PCE (%)
AM 1.5G	100.0	0.545	38.1	74.9	15.6
	7.3	0.455	7.0	66.6	14.3
	11.1	0.476	10.3	67.1	14.5
λ_{660}	24.5	0.500	23.6	70.3	16.6
	49.1	0.516	46.9	71.3	17.2
	69.3	0.518	64.0	71.1	16.7
	3.0	0.425	3.2	63.0	14.0
	4.6	0.437	4.7	65.5	14.3
λ_{809}	7.7	0.462	8.4	68.1	16.8
	12.5	0.478	13.6	70.2	18.0
	24.1	0.508	27.1	71.6	20.1
	49.8	0.531	54.4	70.3	20.0

*: The Si is commercial cell, and the area of the cell is 0.2 cm^2.

Supplementary Table 10. The estimated cost of single concentrating OPV cell with 0.25 cm^2

Cost	0.25 cm^2 concentrating cell (CNY)
Substrate	0.094
MeO-2PACZ	0.0205
perovskite	0.307
C60	0.026
BCP	0.0012
Ag	0.027
Encapsulation	0.027
Total cost	0.316

The PVKs device architecture was ITO/MeO-2PACZ/perovskite/C60/BCP/Ag. The pre-cleaned ITO-coated glass substrates were UV/ozone-treated for 20 min. A 0.5 mg ml^{-1} MeO-2PACZ solution dissolved in ethanol was spin-coated on substrates at 3000 r.p.m. for30 s in a nitrogen glovebox, followed by annealing at 100°C for 10 min. The perovskite composition is $Rb_{0.05}Cs_{0.05}MA_{0.05}FA_{0.85}Pb(I_{0.95}Br_{0.05})_3$, and the initial stock perovskite solution is 1.5 M.

For preparing the precursor solution of perovskite, CsI (19.5 mg), RbI (15.9 mg), MABr (8.4 mg), FAI (219.5 mg), PbI_2 (656.9 mg) and $PbBr_2$ (27.5 mg) were dissolved in 1-ml mixed solvent of DMF:DMSO (v:v = 4:1). Then, the mixture solution was stirred at room temperature for 2-3 h. After the materials are dissolved, the film is deposited on the substrate at a spinning speed of 1000 rpm for 10 s, and at 3000 rpm for 40 s afterwards. After 25 s into the second stage, 150µl chlorobenzene antisolvent was dropped on top of the spinning substrates. The perovskite sample was subsequently annealed at 100℃ for 20 min. Afterwards, samples were transferred to an Angstrom evaporator for C_{60} (30 nm)/BCP (6 nm)/Ag (100 nm) deposition.

Supplementary References

1. Fafard, S. & Masson, D. P. Perspective on photovoltaic optical power converters. *J. Appl. Phys.* **130**, 160901, (2021).
2. Bi, P. *et al.* Reduced non-radiative charge recombination enables organic photovoltaic cell approaching 19% efficiency. *Joule* **5**, 2408-2419, (2021).
3. Gao, F., Tress, W., Wang, J. & Inganas, O. Temperature Dependence of Charge Carrier Generation in Organic Photovoltaics. *Phys. Rev. Lett.* **114**, 128701, (2015).
4. Meng, L. *et al.* Organic and solution-processed tandem solar cells with 17.3% efficiency. *Science* **361**, 1094-1098, (2018).
5. Yoo, S., Domercq, B. & Kippelen, B. Intensity-dependent equivalent circuit parameters of organic solar cells based on pentacene and C60. *J. Appl. Phys.* **97**, 103706, (2005).
6. Hou, J. H., Inganas, O., Friend, R. H. & Gao, F. Organic solar cells based on non-fullerene acceptors. *Nat. Mater.* **17**, 119-128, (2018).
7. Schoen, J. et al. Improvements in ultra-light and flexible epitaxial lift-off GaInP/GaAs/GaInAs solar cells for space applications. *Prog. Photovoltaics* **30**, 1003-1011, (2022).
8. Rath, J. K., Brinza, M., Liu, Y., Borreman, A. & Schropp, R. E. I. Fabrication of thin film silicon solar cells on plastic substrate by very high frequency PECVD. Sol. Energy Mater. Sol. Cells **94**, 1534-1541, (2010).
9. Wu, J. et al. Ultralight flexible perovskite solar cells. Sci. China Mater. **65**, 2319-2324, (2022).
10. Zheng, X. et al. Versatile organic photovoltaics with a power density of nearly 40 W g($^{-1}$). *Energy Environ. Sci.* **16**, 2284-2294, (2023).
