# Supplementary material for: Organic laser power converter for efficient wireless micro power transfer
Source: Nat Commun. 2023 Sep 7;14:5511. doi: 10.1038/s41467-023-41270-1 (PMC10484967; doi:10.1038/s41467-023-41270-1)
Supplement: Supplementary file 3 — Reporting Summary [file 41467_2023_41270_MOESM3_ESM.pdf]

nature research

Corresponding author(s): Jianhui Hou

Solar Cells Reporting Summary

Nature Research wishes to improve the reproducibility of the work that we publish. This form is intended for publication with all accepted papers reporting the characterization of photovoltaic devices and provides structure for consistency and transparency in reporting. Some list items might not apply to an individual manuscript, but all fields must be completed for clarity.

For further information on Nature Research policies, including our data availability policy, see Authors & Referees.

Experimental design

Please check: are the following details reported in the manuscript?

1. Dimensions

Item	Yes/No	Details
Area of the tested solar cells	☒ Yes ☐ No	We focus on 0.037 cm2-area cells with additional results of 20.0 cm-2 cells.
Method used to determine the device area	☒ Yes ☐ No	Device area was determined by optical microscope.

2. Current-voltage characterization

Item	Yes/No	Details
Current density-voltage (J-V) plots in both forward and backward direction	☐ Yes ☒ No	Just J-V plot in forward direction since there is no hysteresis in organic solar cell.
Voltage scan conditions<br>*For instance: scan direction, speed, dwell times*	☒ Yes ☐ No	J–V curves were measured in the forward direction from −0.5 to 1.5 V, with a scan step of 50 mV and a dwell time of 5 ms.
Test environment<br>*For instance: characterization temperature, in air or in glove box*	☒ Yes ☐ No	The cells were measured in glove box. The temperature was fixed at 25 °C during the I-V scans.
Protocol for preconditioning of the device before its characterization	☐ Yes ☒ No	No preconditioning protocol.
Stability of the J-V characteristic<br>*Verified with time evolution of the maximum power point or with the photocurrent at maximum power point; see ref. 7 for details.*	☐ Yes ☒ No	N.A.

3. Hysteresis or any other unusual behaviour

Item	Yes/No	Details
Description of the unusual behaviour observed during the characterization	☐ Yes ☒ No	No. In general, organic solar cells do not have hystereisis problems.
Related experimental data	☐ Yes ☒ No	N.A.

4. Efficiency

Item	Yes/No	Details
External quantum efficiency (EQE) or incident photons to current efficiency (IPCE)	☒ Yes ☐ No	We provided EQE spectra for various cells in Figure 1b and 4c.
A comparison between the integrated response under the standard reference spectrum and the response measure under the simulator	☒ Yes ☐ No	We provided the comparison between the integrated Jsc from the EQE and the Jsc obtained from I-V scan in Table 2.
For tandem solar cells, the bias illumination and bias voltage used for each subcell	☐ Yes ☒ No	N.A.

5. Calibration

Item	Yes/No	Details
Light source and reference cell or sensor used for the characterization	☒ Yes ☐ No	A class solar simulator (Taiwan, Enlitech SS-F5-3A) was used as light source, providing 100 mW cm-2 of simulated AM I.5G irradiation, which was calibrated by a standard silicon solar cell. The illumination intensities of the laser sources were measured by the power meter (S142C, Thorlabs).

Confirmation that the reference cell was calibrated and certified

☒ Yes ☐ No

The standard silicon solar cell was calibrated by National Institute of Metrology (NIM), China

Calculation of spectral mismatch between the reference cell and the devices under test

☐ Yes ☒ No

The light spectrum used for measurements matches well with the reference silicon cell, and we did not calculate the spectral mismatch between the reference cell and the tested devices.

6. Mask/aperture

Size of the mask/aperture used during testing

☒ Yes ☐ No

The aperture area of 0.0223 cm2 were used for testing cells with area of 0.037 cm2.

Variation of the measured short-circuit current density with the mask/aperture area

☐ Yes ☒ No

Our cells were only measured by the mask with fixed area.

7. Performance certification

Identity of the independent certification laboratory that confirmed the photovoltaic performance

☐ Yes ☒ No

N.A.

A copy of any certificate(s)
Provide in Supplementary Information

☐ Yes ☒ No

N.A.

8. Statistics

Number of solar cells tested

☒ Yes ☐ No

More than 5 devices.

Statistical analysis of the device performance

☒ Yes ☐ No

We provided statistical analysis in Table 2 and Supplementary Table 6.

9. Long-term stability analysis

Type of analysis, bias conditions and environmental conditions
For instance: illumination type, temperature, atmosphere humidity, encapsulation method, preconditioning temperature

☒ Yes ☐ No

The devices were encapsulated by exposing epoxy resin under 365 nm UV lamp for 10 min and further tested in air (45% humidity). An array of 660 nm light-emitting diodes (LEDs) was utilized as the light source and the light intensity was 9.46 mW/cm2, which was the same as the best intensity judged by the PCE. During the storage (open-circuit conditions) and measuring, the devices were always kept at 25°C. The area of the device is 0.04 cm2.
